# LACE Index to Predict the High Risk of 30-Day Readmission in Patients With Acute Myocardial Infarction at a University Affiliated Hospital

**DOI:** 10.3389/fcvm.2022.925965

**Published:** 2022-07-11

**Authors:** Vasuki Rajaguru, Tae Hyun Kim, Whiejong Han, Jaeyong Shin, Sang Gyu Lee

**Affiliations:** ^1^Department of Healthcare Management, Graduate School of Public Health, Yonsei University, Seoul, South Korea; ^2^Department of Global Health Security, Graduate School of Public Health, Yonsei University, Seoul, South Korea; ^3^Department of Preventive Medicine, College of Medicine, Yonsei University, Seoul, South Korea; ^4^Institute of Health Services Research, Yonsei University, Seoul, South Korea

**Keywords:** readmission, acute myocardial infarction, risk assessment, prediction, hospital, quality improvement

## Abstract

**Background:**

The LACE index (length of stay, acuity of admission, comorbidity index, and emergency room visit in the past 6 months) has been used to predict the risk of 30-day readmission after hospital discharge in both medical and surgical patients. This study aimed to utilize the LACE index to predict the risk of 30-day readmission in hospitalized patients with acute myocardial infraction (AMI).

**Methods:**

This was a retrospective study. Data were extracted from the hospital's electronic medical records of patients admitted with AMI between 2015 and 2019. LACE index was built on admission patient demographic data, and clinical and laboratory findings during the index of admission. The multivariate logistic regression was performed to determine the association and the risk prediction ability of the LACE index, and 30-day readmission were analyzed by receiver operator characteristic curves with C-statistic.

**Results:**

Of the 3,607 patients included in the study, 5.7% (205) were readmitted within 30 days of discharge from the hospital. The adjusted odds ratio based on logistic regression of all baseline variables showed a statistically significant association with the LACE score and revealed an increased risk of readmission within 30 days of hospital discharge. However, patients with high LACE scores (≥10) had a significantly higher rate of emergency revisits within 30 days from the index discharge than those with low LACE scores. Despite this, analysis of the receiver operating characteristic curve indicated that the LACE index had favorable discrimination ability C-statistic 0.78 (95%CI; 0.75–0.81). The Hosmer–Lemeshow goodness- of-fit test P value was *p = 0.920*, indicating that the model was well-calibrated to predict risk of the 30-day readmission.

**Conclusion:**

The LACE index demonstrated the good discrimination power to predict the risk of 30-day readmissions for hospitalized patients with AMI. These results can help clinicians to predict the risk of 30-day readmission at the early stage of hospitalization and pay attention during the care of high-risk patients. Future work is to be focused on additional factors to predict the risk of 30-day readmissions; they should be considered to improve the model performance of the LACE index with other acute conditions by using administrative data.

## Introduction

In general, cardiovascular diseases (CVDs) are considered a leading cause of unexpected mortality and morbidity and represent a serious public health concern globally (1–3). Hospital readmissions, especially unplanned ones, are costly for the healthcare industry, and readmission frequency is used to judge hospital quality, as unplanned readmission indicates the failure of the initial intervention (4). The 30-day readmission rates are publicly reported and recent health-reform legislation has endorsed the use of readmission rates for hospital profiling in various countries (5). While some efforts have led to a reduction in cardiovascular disease-related readmissions, it has not been possible to recommend these guidelines widely (1, 6–8). The widely accepted common characteristic of cardiovascular disease is the difficulty in curing it once it has developed, due to the structural dysfunction which cannot be differentiated as emergent and non-emergent AMI (9). AMI continues to be a major cause of mortality and re-hospitalization rates and AMI remains high in the Asia-Pacific population. As per the trends in the prevalence of AMI between 2005 and 2018 reported by the Korea Acute Myocardial Infarction Registry (KAMIR), the mean age and gender ratio gradually increased from 66.9 to 78.0% (10). Moreover, the Korean Acute Heart Failure Registry study found a 90-day readmission rate of 8.1%, a 1-year mortality rate of 15%, and 34.6% of 30-day readmissions (11). AMI is also increasing due to the growth of the aging population. AMI may lead to serious complications, require follow-up medical visits, and repeated readmissions may be a difficult experience for patients and their families (11, 12).

The prediction of the risk for 30-day readmissions has been developed by using the HOSPITAL score (13), PARR-30 in the UK (14), and Patient Admissions Prediction Tool (PAPT) (15). The LACE index is one of the most commonly used indices in the US and Canada (16–20). It was first developed by van Walraven et al. (21) to predict the risk of unplanned readmission or death within 30 days after hospital discharge in medical and surgical patients. The model includes the length of hospitalization stay (L), acuity of the admission (A), comorbidities of patients (C), and the number of emergency department visits in the 6 months before admission (E). Scores range from “0” to “19” and those >10 are considered as high risk for 30-day readmission (22). The higher scores indicate a high risk of readmission. This tool is widely used primarily because its simplicity makes it suitable for day-to-day clinical practice (17–25).

Numerous studies have created models that predict 30-day readmissions by using the LACE index for the prediction of the high risk of 30-day readmissions (16–21, 23). The literature on risk prediction of 30-day readmission emphasizes small patient populations (22–25) or specific patient groups such as those suffering from cardiovascular disease (18–20, 23). Very little known about the LACE index in Asian countries (22, 24). However, no study has been conducted to predict hospital readmission by using the LACE index in South Korea.

Risk prediction of 30-day readmission for patients with AMI could be analyzed through a variety of assessment tools ranging from patient interviews to screening methods, by using a different set of variables (26–28). Several studies have investigated the predictors, viz., demographic characteristics, admission and discharge predictors, major surgery, comorbidities, length of stay, medications, and special procedures that are associated with 30-day readmissions (29, 30). One of the first steps in reducing 30-day readmissions is understanding and determining the key causes that lead to instances of readmission and developing a predictive model to assess the risk of readmission. Further, predicting the high risk of 30-day readmission would help avoid unplanned 30-day readmission by enabling targeted interventions.

The specific aim of this study was to use the LACE index to predict the risk of 30-day readmissions in AMI patients after discharge from the hospital because there is no prior study on the prediction of 30-day readmission using the LACE index. This study also aims to assess model performance by identifying patients at risk of 30-day readmission and compare the risk prediction ability relating to 60, 90, and 365 days (1 year) hospital readmissions by using the same LACE index.

## Methods

### Study Design and Setting

A retrospective cohort study design was adopted, and data was derived from January 2015 to December 2019, using the electronic health records of a single university-affiliated hospital in Seoul, Korea. Patients aged 19 years and older, were eligible and hospitalized for AMI as a principal diagnosis and confirmed by using the International Classification of Disease (ICD-10) codes (I20-I25). We excluded patients transferred to other hospitals and those who were not admitted directly from the Emergency Department. We included all patients who were discharged alive from the index hospitalization for the final analysis.

This study protocol was reviewed and approved by the Institutional Review Committee of Yonsei University (4-2021-1047). The ethical consideration of patient consent was waived, and confidentiality was followed by the de-identification of all potentially identifiable data.

### Dependent Variables

Our primary outcome can be defined as hospital readmissions within 30-days for patients diagnosed with AMI as an index of hospitalization. The LACE index score was calculated for each patient, which includes the length of stay (L), acuity of admission (A), comorbidities (C), and emergency visits within the past 6 months. The scoring patterns were calculated and reported in the previous study (16, 17). The length of stay was calculated from the first to the last day of hospitalization and patients admitted to the hospital through the emergency department were identified as acuity of admission, which included patients transferred from the other hospitals through the emergency route. Comorbidities were measured by the Charlson comorbidity index (CCI) score (21), based on the International Classification of Diseases (ICD-10). Emergency visits in the past 6 months were measured, with multiple emergency visits within 24 h being considered as a single visit.

### Independent Variables

The demographic data included patients' age, sex, residence, and insurance types. The discharge data included AMI patients aged 19 and older, those whose sex was considered as either male or female, and those with insurance types such as national health insurance, Medicare, and others. The index of admission types was categorized into three: *via* emergency transfer from other hospitals. The discharge type was normal or with necessary preventive measures and against medical advice or transfer to other facilities such as nursing homes or long-term care centers. In addition, length of stay (LoS), comorbidities by ICD 10 code, primary diagnosis, treatment specialty, admission source, and discharge types were obtained from the hospital EMR data. The 30-day readmission was tracked to identify patients' discharge and readmission history and this report was manually confirmed through chart review.

### Data Analysis

The data were analyzed in three ways. First, we performed the chi-square and univariate comparison between 30-day readmission and no-readmission, frequency, and percentage (*N*, %) for categorical data and mean, standard deviation (M, SD) for continuous variables of laboratory data. Second, a multivariate logistic regression analysis was performed to identify the factors associated with 30-day readmissions with Odds Ratio (OR; 95% CI). Third, the prediction ability of the LACE index score was calculated and ROC curves were performed to assess the sensitivity and specificity of the C-statistic prediction model, which ranged from 0.5 (Low discrimination) to 1.0 (Good/high discrimination); it was measured by the area under the curve (AUC). In addition, the Brier score was calibrated to evaluate the accuracy of predicting the risk of 30-day readmission, the values ranging between “0.0” (Perfect accuracy) and “1.0” (perfect inaccuracy). Finally, we investigated a suitable numerical threshold by fitting a logistic regression model for each outcome with dichotomized LACE scores above and below specific thresholds, using sensitivity, specificity, ORs (95% CI), and C-statistics (95% CI) on each outcome's respective receiver operating characteristic (ROC) curve and compared the same with the 60-, 90-, and 1-year readmission as a secondary analysis. *p*-values < 0.05 were considered statistically significant. Statistical analysis was performed using SAS 9.4 (SAS Institute Inc., Cary, NC, USA).

## Results

### Study Participants

**Figure 1:**
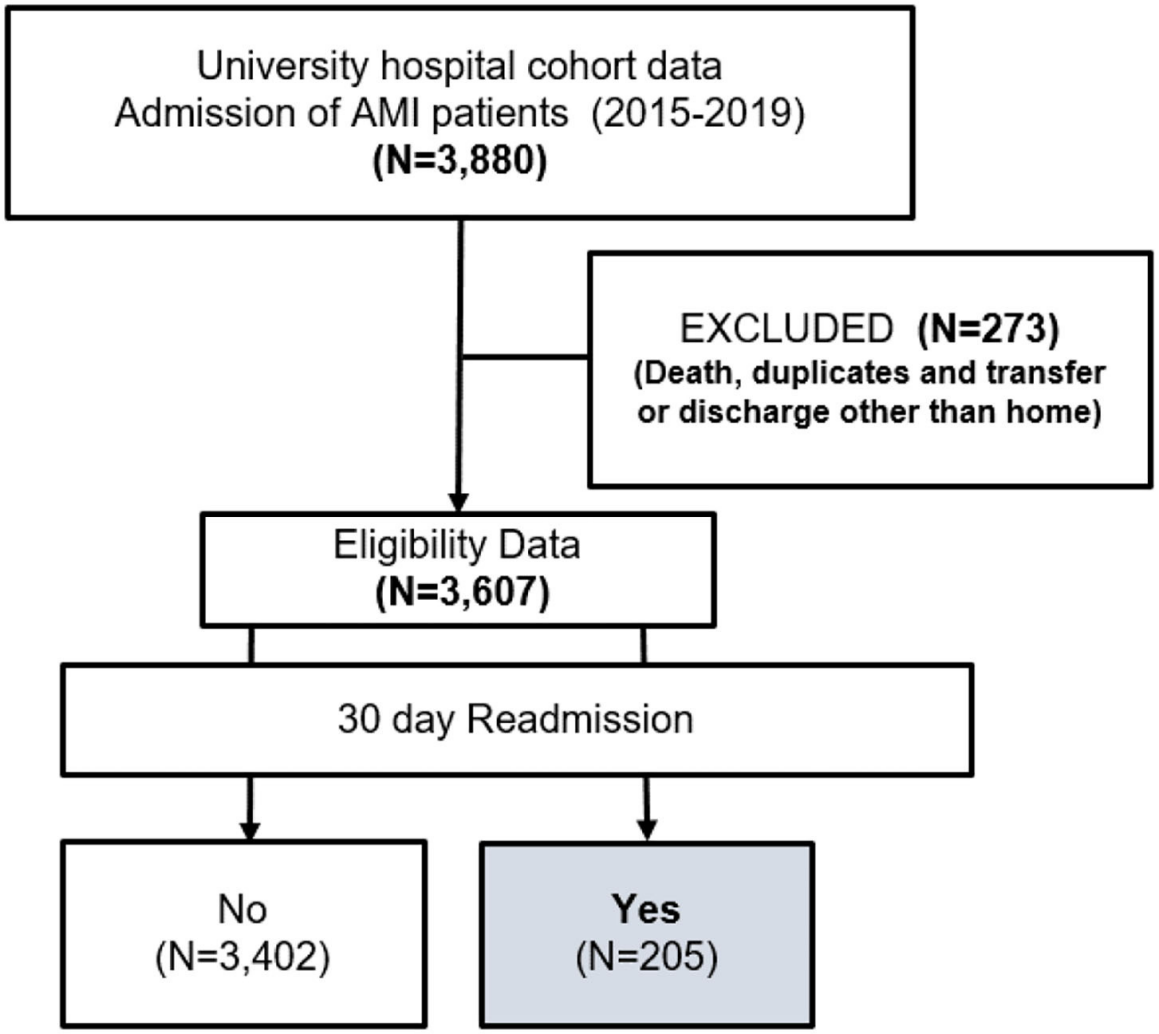
Flow chart for selection process of study population.

The study cohort included a total of 3,607 patients, of whom 205 reported 30-day readmissions among patients hospitalized with AMI during the study period (Figure 1).

### Characteristics of the Patients With 30-Day Readmission vs. Non-Readmission

Table 1 summarizes the observed frequency (percentage) and mean (standard deviation) baseline data of 30-day readmissions and non-readmissions. More than half the patients were male (58.5%), in the age group of over 65 years (57.1%). Most of them resided in Seoul city (77.1%) and had national health insurance membership (55.6%). The length of stay (LOS) was about 3 days (39.5%), and those admitted through emergency department visits formed 51.7%, while those with two comorbidities (38%) showed 30-day readmissions. Laboratory findings revealed that patients readmitted within 30-days had lower hemoglobin levels (10.6 ± 9.3; *p* < 0.001) which was significant. However, there were no statistical differences in any other laboratory findings.

**Table 1:** Baseline characteristics of 30-day readmission vs. no readmission patients admitted with AMI.

**Variables**	**Characteristics**	**30-day readmission**				
		**Yes (*****n*** **=** **205)**		**No (*****n*** **=** **3,402)**		***p***
		***N***	**%**	***N***	**%**	
LACE index score	0–4	42	20.5	838	24.6	<0.001
	5–9	71	34.6	1771	52.1	
	≥10	92	44.9	793	23.3	
Age (years)	<34 years	12	5.9	591	17.4	<0.001
	35–64	76	37.1	896	26.3	
	≥65	117	57.1	1915	56.3	
Sex	Male	120	58.5	1,928	56.7	<0.001
	Female	85	41.5	1,474	43.3	
Residence	Seoul (capital area)	158	77.1	2,163	63.6	<0.001
	Metropolitan cities	47	22.9	905	26.6	
	Other cities	0	0.0	334	9.8	
Health insurance	NHI	114	55.6	2,796	82.2	0.008
	Medicare	86	42.0	486	14.3	
	Others	5	2.4	120	3.5	
Length of stay	≤2	49	23.9	795	23.4	
	3	81	39.5	908	26.7	
	4	22	10.7	708	20.8	0.114
	5	24	11.7	622	18.3	
	6	18	8.8	221	6.5	
	≥7	11	5.4	148	4.4	
Admission Route	ER	106	51.7	2,191	64.4	<0.001
	Transfer from other hospital *via* ER	99	48.3	711	35.6	
Comorbidities (CCI score)	1	68	33.2	1,865	54.8	0.023
	2	78	38.0	1,023	30.1	
	≥3	59	28.8	514	15.1	
Laboratory findings (M ± SD)	SBP (mmHg)	125.1 (15.6)		120.8 (17.5)		0.191
	Hemoglobin, mg/dL	10.6 (9.3)		11.4 (9.8)		<0.001
	WBC, ×10^3^/UL	3.6 (1.1)		5.8 (3.0)		0.441
	Platelet, ×10^3^/μL	223.1 (99.8)		225.6 (111.8)		0.418
	Creatinine, mg/Dl	1.65 (2.4)		1.2 (1.1)		0.541
	Potassium, mmol/L	3.9 (0.5)		4.04 (3.2)		0.842
	Sodium, mmol/L	137.2 (4.5)		139.5 (4.1)		0.691
	Estimated GFR (mL/min/m^2^)	39 (25.8)		41 (28)		0.511
Discharge type	Normal	38	18.5	2,988	87.8	0.121
	Others*	167	81.5	414	12.2	

*N (%), number (Percentage); M (SD), Mean ± standard deviation; p-value, chi-square test; NHI, National health insurance; ER, Emergency route; CCI, Charlson comorbidity index (1, 2, ≥3 represent the number of comorbidities); WBC, White blood cell; GFR, Glomerular filtration rate; OP, Outpatient;*

* *Home with support services, transfer to long-term care/other institution, Left against medical advice*.

### Multivariate Logistic Regression Analysis for 30-Day Readmission in Patients Hospitalized With AMI

In a multivariate logistic regression analysis, risk factors determined to be independently associated with 30-day readmissions are shown in Table 2. Older patients aged <65 years (OR, 8.15; 95% CI, 4.07–6.24), who were male (OR, 1.07; 95% CI, 1.06–1.07), had Medicare insurance (OR, 1.07; 95% CI, 1.00–1.11), admitted through the emergency route (OR, 1.45; 95% CI, 1.42–1.54), and belonged to the other discharge types (OR, 1.09; 95% CI, 1.04–1.14) were more likely to have 30-day readmission, after controlling potential confounders. In addition, LACE index risk scores ≥ 10 (OR, 2.71; 95% CI, 1.03–4.37) were highly associated with 30-day readmission than lower LACE scores (0–4 and 5–9).

**Table 2:** Multivariate logistic regression analysis for 30-day readmission in patients hospitalized with AMI (*N* = 205).

**Variables**	**Characteristics**	**30-day readmissions (yes)**			
		**OR**	**95% CI**		***p***
Age, years	19–44	1.00			
	45–64	3.15	0.86	6.17	0.118
	≥65	5.15	4.07	6.24	<0.001
Sex	Male	1.07	1.06	1.07	<0.001
	Female	1.00			
Health insurance	NHI	1.00			
	Medicare	1.07	1.00	1.11	0.003
	Others	0.98	0.85	1.13	0.441
Admission route	ER	1.45	1.42	1.54	0.021
	Transfer from other hospital *via* ER	1.00			
Discharge type	Normal	1.00			
	Others*	1.09	1.04	1.14	<0.001
LACE index_score	0–4	1.00			
	5–9	1.13	1.11	1.15	0.007
	≥10	2.71	1.03	4.37	0.010

*OR, odds ratio; CI, confidence interval; AMI, Acute myocardial infarction; IQR, Interquartile range; NHI, National health insurance; ER, Emergency visit; op, Outpatient visit*;

* *Home with support services, transfer to long-term care/other institution, Left against medical advice*.

The association between the different LACE variables was found to highly predict the risk of 30-day readmission; the length of stay (OR, 2.01; 95% CI, 1.35–2.98), index of admission (OR, 1.21; 95% CI, 1.01–1.44), comorbidity (OR, 1.72; 95% CI, 1.16–2.55), and the number of emergency visits in the last 6 months (OR, 1.61; 95% CI, 1.14–2.52) were statistically significant at *p* < 0.001 level.

### Sensitivity Analysis

**Figure 2:**
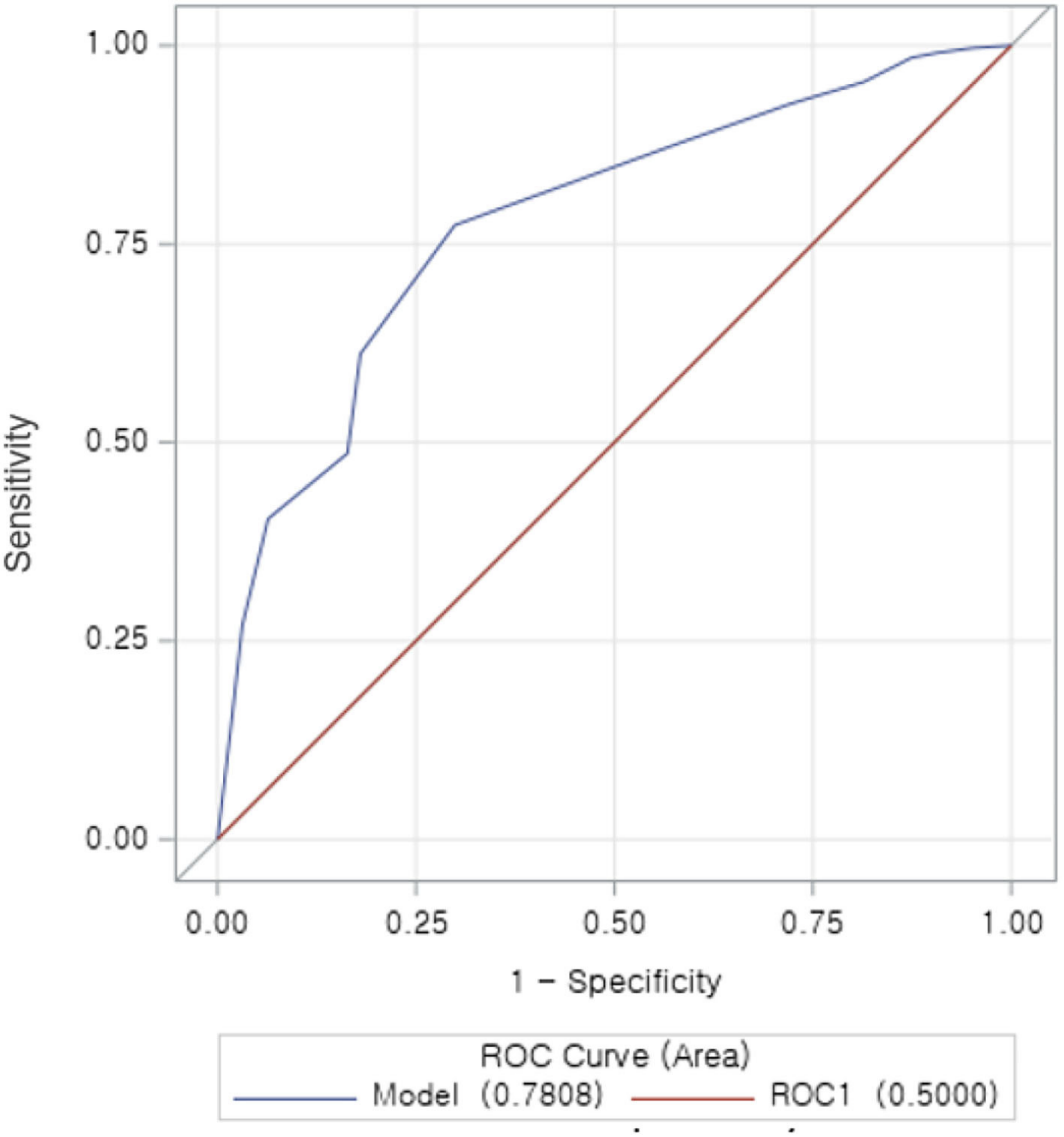
Receiver operator characteristic (ROC) curve for the LACE index in hospitalized AMI patients. The ROC curve illustrates the risk prediction for 30-day readmission at different cutoff points. With increased sensitivity and decreased specificity. The area under the curve (AUC), which is equal to the *C*-statistic (0.78), indicating a favorable model to predict the risk of 30 days readmission in patients hospitalized with AMI.

**Figure 3:**
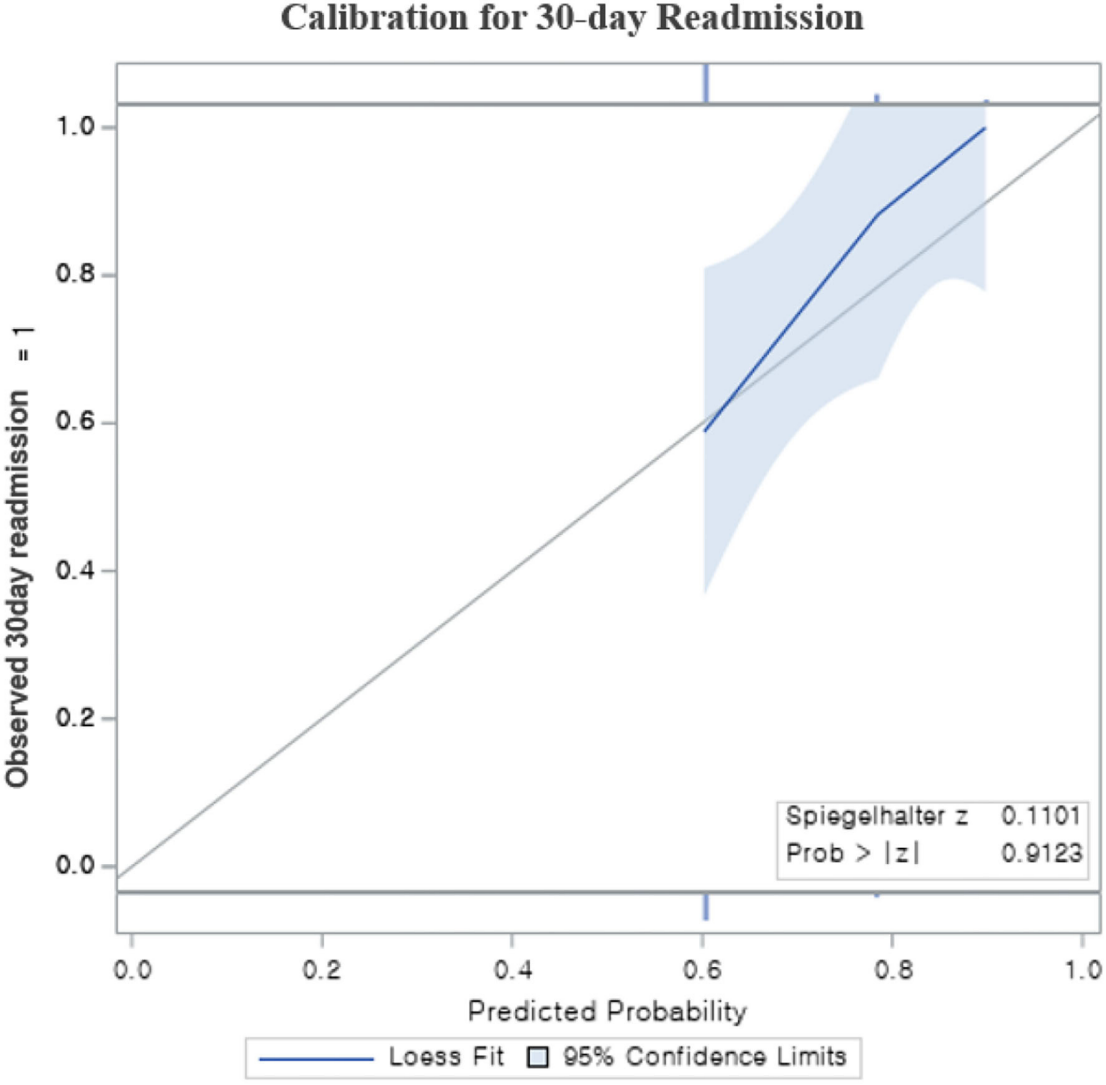
The calibration plot for Risk prediction model for 30-day readmission; The plot contains a gray diagonal line, which represents perfect calibration. The light blue band is a 95% confidence, calibration plot of this fit also be close to the diagonal. Calibration plot, Hosmer–Lemeshow plot; *p* = 0.912.

The discrimination ability of the model for risk prediction of 30-day readmission in Figure 2 shows a modest performance of the LACE index in risk prediction for 30-day readmission with a C-statistic of 0.78 (95% CI 0.75–0.81). The ROC analysis outcome of 30-day readmissions is shown in an AUC curve (Figure 2). The Brier score for the LACE score in this setting was 0.042, indicating overall good performance and the Hosmer-Lemeshow goodness-of-fit test *p*-value was *p* = 0.920, indicating that the model was well-calibrated; this was consistent with the calibration plot (Figure 3). These findings indicated that the LACE index model has a favorable risk prediction ability for 30-day readmission of patients hospitalized with AMI.

### Secondary Analysis

**Figure 4:**
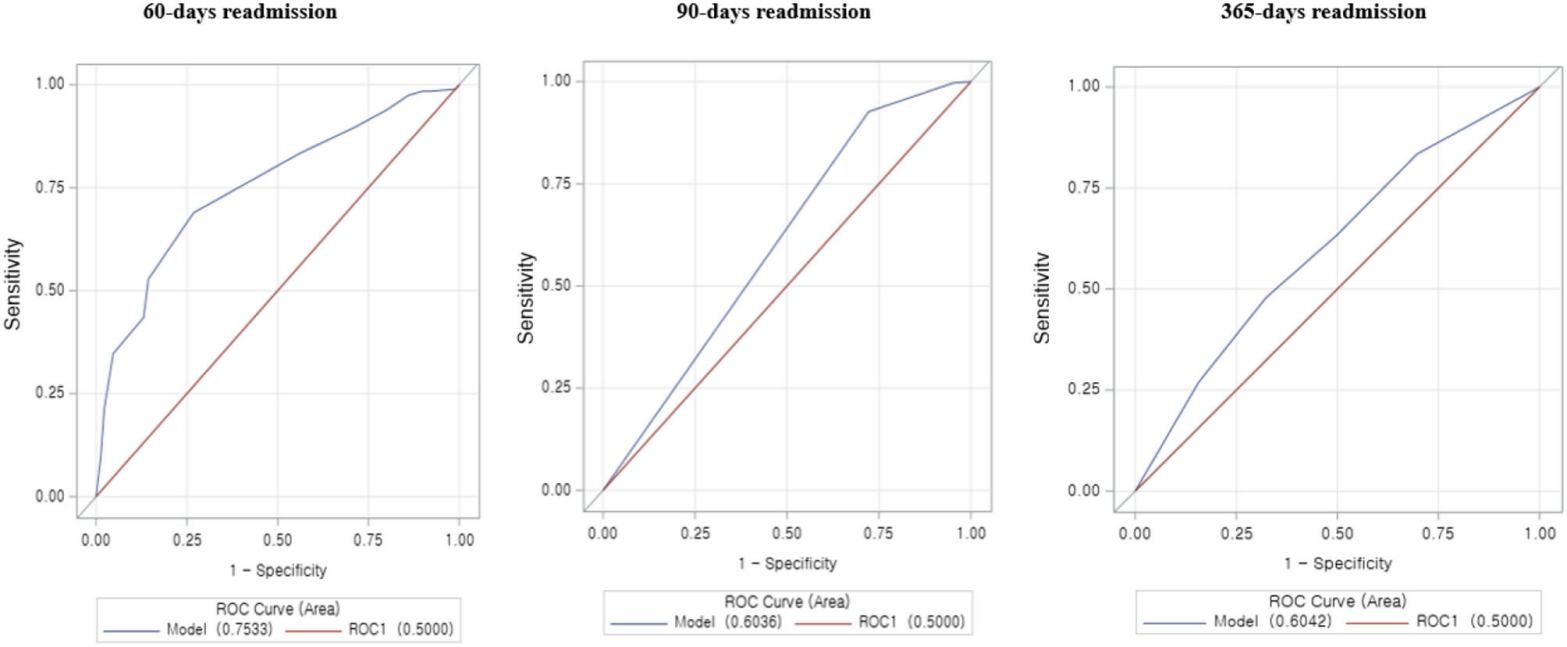
An area under the receiver operator curve (ROC) for the LACE index predict 30-day readmission in hospitalized patients with AMI. The ROC curve illustrates the risk prediction for 60, 90, and 365 days (1 year) readmission at different cutoff points.

Figure 3 illustrates the frequency distribution of readmissions based on the time duration calculated as a secondary analysis. In addition, a new prediction model analysis of the LACE index was performed with different combinations of readmissions for 60 days, 90 days, and 1 year as shown in Table 3, as it was not relevant to our present cohort study. However, these findings were varied in the prediction ability of the LACE index; the C-statistic for each model of readmissions was: 60 days = 0.75 (95% CI, 0.71–0.79), 90 days = 0.60 (95% CI, 0.58–0.62), and 1 year = 0.60 (95% CI, 0.56–0.64). The results demonstrated that the LACE index is better in predicting the risk of 30- and 60-days readmissions than 90 days and 1-year readmissions (Figure 4).

**Table 3:** Secondary analysis of comparison between 30-day readmission and 60-, 90-, and 365-days (1 year) readmissions of patients hospitalized with AMI.

**Hospital readmissions**			
	**60-days**	**90-days**	**365-days (1 year)**
AUC	0.75	0.60	0.60
95% CI	0.71–0.79	0.58–0.62	0.56–0.64
*p*	<0.001	<0.001	<0.001

*AUC, Area under the curve*.

## Discussion

This study aimed to predict the risk of 30-day readmissions by using the LACE index score and validated models for patients hospitalized with AMI. A systematic review was retrieved from 16 unique LACE index articles to predict the risk for 30-day readmissions in specific diseases and population groups in limited countries prior to this study; there were no such studies found in South Korea. The overall 30-day readmission rate was lower than the reported 15.5–15.9% (7, 26, 27). However, it is difficult to compare the studies directly because the published studies used Medicare's fee-for-service claims data in the US and included only elderly Medicare patients. In addition, the variation and internal protocol in hospitals' systems across the nation could account for the changes in the 30-day readmission rate. Our study found that men were 13% more likely to have 30-day readmission than women. This is similar to an earlier retrospective study conducted in patients with heart failure, COPD, and all-cause readmissions predicted by using the LACE index (17–20, 23, 24).

We also found that patients discharged for other reasons such as against medical advice or voluntarily discharged were more prone to 30-day readmissions, compared to those discharged normally. This finding is consistent with other studies (8–12) and the performance of the LACE index was found to vary with disease conditions (16–19). However, patients' discharge destination showed that those discharged directly to their homes were at a greater risk of 30-day readmission than those transferred to inpatient rehabilitation or other care, including home care service. This concurred with another study (9), in addition to another version of a competing risk issue previously mentioned. An alternative interpretation is that patients discharged home are less likely to die before readmission than those discharged to Skilled Nursing Facilities (SNFs). Patients discharged to SNFs can also, to some extent, get the care they need in the case of a potential exacerbation of their underlying condition, diverting some potential readmissions, whereas patients discharged home may not be able to access that care as easily, without going to the hospital. This would also depend on which post-discharge case management programs are in place. Therefore, post-discharge interventions and resources are required for the patients who are discharged directly to their homes, as it would help in preventing or reducing 30-day readmissions.

A significant finding of this study was that 30-day readmissions were predominantly related to socioeconomic factors, rather than clinical findings of the index of admission. This was consistent with other studies with different disease conditions, where the clinical findings had least or not been considered (5, 8, 10–12, 14, 16–19, 27). Therefore, the prediction of readmission for acute care suggests that the attention to clinical findings would be considered in long-term care than in acute care settings. Similar to other reports on CVD-related 30-day readmission rates among Medicare beneficiaries, this study found higher 30-day readmission rates among men, patients aged < 65 years, those from lower-income households, and those with multiple comorbidities (12, 18–20, 26, 27, 30).

Hence, it is hypothesized that fluctuations in emergency room visit trends could be a possible cause of the variations in readmission observed in the later years. Studies have reported various factors contributing to 30-day readmission, including complications of inpatient treatment, irrelevant coordination of care, inferior quality of care, ineffective medication advice, discharge education, and follow-up (8, 28, 29). In contrast to the LACE index, the length of stay and acuity of admission were not associated with the risk of 30-day readmission, after adjusting the covariates in the multivariate logistic regression model. It is possible that the duration of admission was affected by other factors such as demographic characteristics and did not reflect the severity of illness entirely in this cohort study.

Our previous literature review identified that several other factors such as age, comorbidity index, and emergency department visits in the past 6 months were significant in the prediction of 30-day readmission risk (21). In all the predictive models, AMI was statistically significant. This is altogether found in the chronic nature of readmissions among patients with cardiovascular diseases (18–21, 23, 29). Our study found results from 2015 to 2019 with an absolute possibility of risk prediction for 30-day AMI readmissions with a LACE score of more than 10. The studies of Medicare insurance have suggested similar results, that implementation of the LACE index is associated with a decrease in cardiovascular disease readmissions. Some studies have reported a decline in 30-day readmissions after the LACE index implementation phase (18–25).

The LACE index allows clinicians to calculate an individual's unique risk of 30-day readmission quickly and accurately, enabling improved coordination of care between healthcare professionals and the implementation of various strategies to prevent readmissions among high-risk patients. Reducing readmissions not only reduces healthcare expenditure but most importantly, also improves patient outcomes and satisfaction. Readmissions are not only inconvenient and costly to the patient but also come with inherent risks such as hospital-acquired infections, which impact patient outcomes negatively. Therefore, this study suggests using the LACE index, as it would be helpful for physicians to make better clinical decisions about the duration and aggressiveness of patient treatment and management and for curtailing premature discharge for patients with high readmission risk.

## Conclusion

We have used the novel findings of an important tool the LACE index with associated factors—to predict the 30-day readmissions, for the first time in South Korea. LACE can be computed without the aid of special software and does not require complex information such as community-specific rates of admission or economic status. Given its ease of use at the bedside, LACE is commonly applied to risk-stratify patients hospitalized with medical illnesses. Therefore, focusing on the LACE index is recommended to predict the risk of 30-day readmissions as it is critical for reducing the future readmission burden of patients with acute CVDs. In addition, constant follow-up of the AMI patients may also be needed to reduce the readmission risks of those directly discharged to their homes. The findings of this study would be communicated to healthcare managers so that they can implement policies to use the LACE index to easily predict the risk of early readmissions and avoid unnecessary medical expenditure. The findings will assist in targeting future interventions to predict 30-day readmissions and should be expanded by using national administrative data that includes prospective design, more periods with all the causes of 30-day readmissions, and additional factors, to get a better understanding of the association between 30-day readmissions and cost-effectiveness analysis by using the LACE index and to demonstrate the lag effects of readmission rates on operating margin.

## Limitations

This study has some limitations. First, the patients were selected from a single hospital in the metropolitan city, and its findings are not intended to be generalized to other areas in Korea. Further work is needed to characterize whether certain ICD-10-AMI codes represent 30-day readmission that could be prevented through improved clinical-based care or healthcare systems. Moreover, our study was designed with observations and used the retrospective cohort data of individual hospital data, including laboratory data. Causation must be considered for generalizing the findings, as there might be unnoticed variables of laboratory data as confounding variables. Second, the cohort data of patients hospitalized with AMI between 2015 and 2019 was unique and changes were made to clinical guidelines, in particular to the provision of acute services after the introduction of cardiovascular conditions. We did not focus on the spectrum of NSTEMI and STEMI. In particular, we do not know the baseline and the treatment that will be initiated in post-discharge management. Time-varying covariates do not yield the perfect prediction model, which limits the prediction of 30-day readmission risk in some extended clinical management in treatment (31). We were not able to address the details of the TRIPOD statement (32). However, we have included the TROPID checklist as a Supplementary Material in this study. Therefore, these issues should be addressed in future studies for better implementation of the predictive model for further consideration. However, these results remain novel because, for the first time, reliable data has been offered on 30-day readmissions after the hospital discharge of patients with AMI in Korea.

## Data Availability Statement

The raw data supporting the conclusions of this article will be made available by the authors, without undue reservation.

## Ethics Statement

The studies involving human participants were reviewed and approved by Yonsei University (IRB: 4-2021-1047: 002). The patients/participants provided their written informed consent to participate in this study.

## Author Contributions

VR, TK, and SL: conceptualization. JS, VR, and WH: data curation and formal analysis. VR and JS: investigation. JS, VR, WH, SL, and TK: methodology. SL and TK: resources and supervision. VR: writing—original draft. JS, SL, and TK: writing—review and editing. All authors contributed to the article and approved the submitted version.

## Conflict of Interest

The authors declare that the research was conducted in the absence of any commercial or financial relationships that could be construed as a potential conflict of interest.

## Publisher's Note

All claims expressed in this article are solely those of the authors and do not necessarily represent those of their affiliated organizations, or those of the publisher, the editors and the reviewers. Any product that may be evaluated in this article, or claim that may be made by its manufacturer, is not guaranteed or endorsed by the publisher.
